# Functional Properties of Nonwovens as an Insulating Layer for Protective Gloves

**DOI:** 10.3390/polym15030785

**Published:** 2023-02-03

**Authors:** Dunja Šajn Gorjanc

**Affiliations:** Department of Textiles, Graphics Art and Design, Faculty for Natural Sciences and Engineering, University of Ljubljana, Snežniška 5, 1000 Ljubljana, Slovenia; dunja.sajn@ntf.uni-lj.si; Tel.: +386-1-200-32-20

**Keywords:** nonwoven fabrics, protective gloves, mechanical properties, elastic properties, air permeability

## Abstract

The basic intention of the present work is to analyze the influence of the incorporated microporous membrane and the technology of the needling process on the functional properties of nonwovens designed as an insulating layer for protective gloves for protection against high temperatures. The investigated nonwovens are produced in carded nonwoven formation and mechanically bonded with needle bonding. The studied nonwovens contain a microporous membrane of polyester (PES) with a thickness of 20 µm (samples marked as ST and STL). In the theoretical part of the research work, the nonwovens and some technology stages are presented. The experimental part of the present work deals with the mechanical properties: breaking stress and strain, viscoelastic properties (yield strength, elastic modulus) and elastic recovery after cyclic loading and thermal conduction. In the experimental part, permeability properties (water vapor permeability, air permeability) are also analyzed. The results of the investigation show that the samples marked as ST and STL, which contain a microporous PES membrane, have a higher breaking stress than the samples marked as T and TL without the microporous PES membrane. Samples marked as ST and STL also exhibit higher values of elongation at break and limit of recoverable deformation (stress and strain at yield) and a lower modulus of elasticity than samples marked as T and TL. The samples marked as ST and STL are mechanically bonded to the lamellar plate using forked needles and therefore have a textured (ribbed) shape that affects the improved mechanical properties. The TL and STL samples, which contain a microporous PES membrane, have higher elastic recovery and lower air permeability than the T and TL samples, while water vapor permeability is lower only for the ST sample.

## 1. Introduction

Research is being conducted on the properties of nonwoven fabrics for protective gloves for protection against high temperatures. The nonwovens are produced by a dry laying process (carding process) and mechanically bonded with needling. A protective film (microporous membrane made of polyester, 20 µm thick) is applied to some of the samples investigated.

The influence of the inserted protective membrane on the functional properties of the nonwoven is investigated.

In the theoretical part, the nonwovens, the classification of nonwovens, the web formation of nonwovens, the web bonding of nonwovens and the finishing are presented, as are the mechanical and elastic properties and the insulation and permeability properties of the nonwoven for work protective gloves.

The main mechanical properties include breaking stress and elongation, highly elastic properties (yield strength and modulus of elasticity) and elastic recovery after cyclic loading, and thermal conductivity and permeability properties: water vapor permeability and air permeability.

The inner (insulating) layer of the protective glove is usually made of nonwoven fabrics, which are usually produced using a dry laying process (carding) and mechanically bonded (by needling).

Protective gloves must provide mechanical protection (abrasion resistance, resistance to deformation during use), thermal protection (protection against high and low temperatures), protection against radiation and protection against chemicals.

The nonwoven fabrics for protective gloves investigated in this study are used as the inner layer of protective gloves for protection against high temperatures during work [1,2,3,4,5,6,7,8,9,10,11,12,13,14,15].

## 2. Theoretical Section

### 2.1. Technology Process of Web for Insulating Layer for Protective Gloves 

Dry-laid, needle-bonded nonwovens for protective clothing account for 40% of total nonwoven production [1,2,3,4,5,6]. In addition to protective clothing, such nonwovens are also used in the construction industry (insulation), the automotive industry (fillers), the textile and footwear industry (fillers, shoe linings, artificial leather, etc.) and the furniture industry (interior design).

The manufacturing process of nonwovens consists of web formation, web bonding and finishing. There are three main nonwoven web formation processes, namely dry laying, wet laying and extrusion processes, and three main web bonding processes, namely mechanical, thermal and chemical bonding processes [1,2,3,4,5,6].

Nonwovens can be produced by extrusion processes, including spun bonding, melt blowing and electrospinning processes. Spunbond nonwovens and melt-blown nonwovens both have larger fiber diameters than electrospun nonwovens. The spun bonding process produces fiber diameters of 10–35 µm, while melt blowing often produces fibers of 1–5 µm, but they can be 300–500 nm. However, electrospinning can produce fibers with a large diameter range from 2 micrometers down to a few nanometers [7].

Electrospinning is one of the processes used to produce nonwovens from nanofibers, along with dry laying, wet laying, and extrusion process. Electrospinning involves spinning nanosynthetic fibers from polymer solutions and melts. Unlike conventional fiber spinning processes, which can produce fibers with diameters down to the micrometer range, electrostatic spinning or electrospinning is capable of producing fibers in the nanometer diameter range or “nanofibers”. In electrospinning, electrostatic forces are used in addition to mechanical forces to drive the fiber formation process.

The electrospun nanofibers provide a very large surface area/mass due to their small diameter, so nonwovens with small fibers can be used for the filtration of partial particles in the separation industry. They can also be used for the adsorption of biological and chemical warfare gases; for protective clothing; in the medical industry as artificial blood vessels, sutures, surgical face masks, fiber-reinforced materials and monodirectional composites; and in agriculture for pesticide control [1,2,3,4,5,6,7].

For protective gloves for protection against high temperatures during work, the inner insulating layer is usually produced by a dry laying process (carding) and mechanically bonded (with a needle).

#### 2.1.1. Dry Laying Process

In the dry laying process, the web is made of staple fibers. These fibers are laid directly from a card to form a web with a basis weight of 10–2500 g·m^−2^. The used carding machines used are not the usual ones; they are equipped with working and stripping rollers. There are three methods of laying a web of fibers.

The basis weight of carding web is usually too low to be used directly as an insulating layer. For the production of heavier carded webs with a basis weight of 80 to 1000 g·m^−2^, a horizontal cross lapper is obligatory in the process line. The “profile” cross lapper using the so-called “ProDyn system” allows for thinner installation of the curtain at the edges and tighter installation of the ceiling across the width of the installation strip.

The programmed positioning of the web using the sensor control continuously regulates the speed of the upper and lower carriage during the laying of the web across the width of the connecting strip, which enables profiled laying of the web in a layered web of plano-convex shape [1,2,3,4,5,6,7].

#### 2.1.2. Web Bonding

The web after laying is mechanically bonded by vertical needling.

Concerning the intensity of needling, we know the following:

- Pre-needling;
- Needling.

By pre-needling and needling we bonded layered web to the final nonwoven fabric. The layered web is lightly cured on the forearm by interlacing the fibers in the web and is ready for drawing in the production process.

The web is first needled with needles inserted on one or both sides. With the help of teeth, they grip the fiber bundles and change their position. For a high-quality prelude, we have to use the teeth to feed the veneer as evenly as possible.

The prelude is followed by the needling phase with the needle. The web is guided into the needling by a pair of feed rollers.

A needle board with a plurality of serrated needles performs a translational movement towards the base plate on which a reinforcing layer is placed, allowing the needles to pierce the web in a vertical direction between the perforated board and the base board. After appropriate consolidation of layered nonwoven textiles, it is also possible to produce structured or textured nonwoven textiles.

For the production of structured nonwoven textiles, it is necessary to use the following:

- A needle board with a fork or special needle instead of a toothed needle;
- A flat base board with a lamellar or brushed base board [1,2,3,8].

For structured needlework, pre-needled nonwovens are fed into a needle space. The needle penetrates the cured net textile vertically with fork needles, the fork needles push textile fiber bundles onto the back of the web and form a one-sided loop structure. The height of the pushed-out loops is adjusted by moving or offsetting the lamellar board to the fixed pick-up plate and by the depth of the needle penetration. For the double-sided loop structure, the needle holder has a laminated base and recording board and fork needles for piercing the front and back of the loop style [1,2,3,4,5,6,7,8].

Needle bonding is a nonwoven process in which fibers are mechanically entangled by repeatedly piercing a preformed dry nonwoven fiber web with needles to produce a nonwoven fabric. The machine that performs this process is called a needle loom. The nonwoven fabric, which is not bonded and is therefore thick and bulky, is fed into the machine by a pair of feed rollers.

It then enters the working area of the machine and passes between a pair of perforated plates. The needles are arranged in rows widthwise on a needle board. The needle board is mounted on a beam which is set in an up-and-down motion by an eccentric crank mechanism. On the down stroke, the needles travel through the perforations of the upper base plate, through the bar and through the perforations of the lower base plate. On the upstroke, the barbed needles retract upward and the base plate strips the web from the needles. This mechanically interlocks the fibers, providing mechanical strength. The needle-bonded nonwoven is discharged by a pair of discharge rollers.

To produce a surface texture with a velour or ribbed effect, the lamellar base plate and the fork needles are used. Fork needles carry tufts of fibers in slatted bars that extend from the entrance to the exit of the lamellar base plate. These fork needles carry large fiber tufts into parallel flap bars. These bars carry the fiber tuft from the input to the output side of both needle boards which are also called the needle loom [1,2,3,4,5,6,7,8].

## 3. Materials and Methods

### 3.1. Materials

The samples studied represent a three-layered material, with the inner layer (insulating layer) consisting of a nonwoven web produced by the carding process (dry laying process) and bonded mechanically (needle-bonded). The web in samples marked as T and TL is bonded on the flat base plate (classic base plate), while the web in samples marked as ST and STL is bonded on the lamellar base plate and has a structured surface. The upper (1st layer) and lower layers (3rd layer) of the samples are made of warp-knitted fabric, and the upper layer is knitted plush made of flame-resistant cotton (FR cotton) fibers. The lower layer (3rd layer) of samples T and TL is made of FR cotton and polyethylene terephthalate (PET) tricot knitted fabric with an inserted weft, while that of samples marked as ST and STL is made of flat warp-knitted fabric from FR cotton and polyethylene terephthalate (PET fibers). The inside (2nd layer) of all the analyzed patterns consists of a nonwoven web, where the web marked with T and TL consists of a mixture of polyethylene terephthalate, viscose and wool fibers (PET/CV/WO) and has a smooth surface, while the fiber of the nonwoven web marked as ST and STL consists of 100% polyethylene terephthalate (PET) fibers and has a structured surface (Table 1). The samples marked as TL and STL also contain a microporous membrane of polyester (PES), which is 20 µm thick (Table 1). The structural properties of analyzed samples are listed in Table 2, while the microscopic view of the samples is presented in Table 3. 

The samples at 70× magnification are listed in Table 3.

### 3.2. Methods

#### 3.2.1. Breaking Force and Elongation

Nonwovens are subjected to different stresses or forces both during processing and later in use. The strength of materials is defined according to the direction of the external force. For textile materials, tensile strength is important. In the tensile test, the tensile force and the tensile elongation are determined, and the tensile force acting on the test sample in the direction of loading at the moment of break and the change in length of the test piece are determined [15,16,17,18,19,20,21,22,23].

The tensile properties were measured on an Instron 5567 dynamometer (Instron, Bristol, UK) according to ISO 13934-1 [17], which can be used to measure the tensile properties of various materials (textiles, paper, cardboard, polymeric materials). The measured values were processed using the program Blue Hill (Instron, Bristol, UK), which is adapted for this type of measurement and gives us an indirect insight into the internal changes and events in the material structure that took place during the tensile test. At the same time, the program also allows us to interpret the measured values later.

The viscoelastic properties of researched samples were determined graphically based on the analysis of the stress/elongation curves of the samples analyzed [18,19,20,21,22,23,24].

#### 3.2.2. Elastic Recovery after Compressive Loading

**Figure 1:**
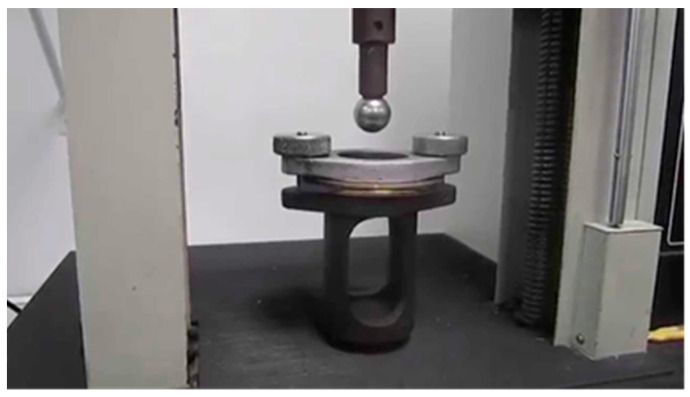
Illustration of the compressive loading of the specimen with a sphere.

The elastic properties of the specimens studied were measured according to the modification of the ASTM 6614 [25] standard, in which the specimen was subjected to a compressive load with a sphere of diameter d = 3.0 cm. The diameter of the specimen holder was 3.5 cm (Figure 1). In this standard, the specimen is loaded with a mass of 1.814 kg or force of 17.8 N and left at this load for 5 min, then lowered to the initial position and then relaxed (5 min). A control cycle is then performed so that the continuous strain can be read and the elastic recovery *E_e_* can be calculated from it (Equation (1)) [15,19].

(1) $$E_{e}=\frac{\varepsilon_{e}}{\varepsilon }\cdot 100$$

where *E_e_* is the elastic recovery (%), *ε_e_* is the elastic strain (%) and *ε* is the total strain (%).

#### 3.2.3. Air Permeability

A very important property of fibers that significantly affects comfort is air permeability. We used the method according to the standard ISO 9237 [26].

Air-Tronic (Mesdan S.p.A., Brescia, Italy) is a device for determining air permeability in terms of air flow rate passing through a sample in a vertical direction under previously known and well-defined conditions. It is used for woven fabrics, knitted fabrics, nonwoven fabrics, technical fabrics for technical purposes, artificial leather, felt, velvet and paper. The machine is additionally equipped with a printing unit. The air flow rate Q, in L/h, was determined on the apparatus. The test area of the sample was 10 cm^2^ and the pressure was 100 Pa.

#### 3.2.4. Thermal Conductivity

Thermal conductivity is used to measure the specific heat transfer through a nonwoven fabric. We measured according to the standard DIN 52612 [27].

**Figure 2:**
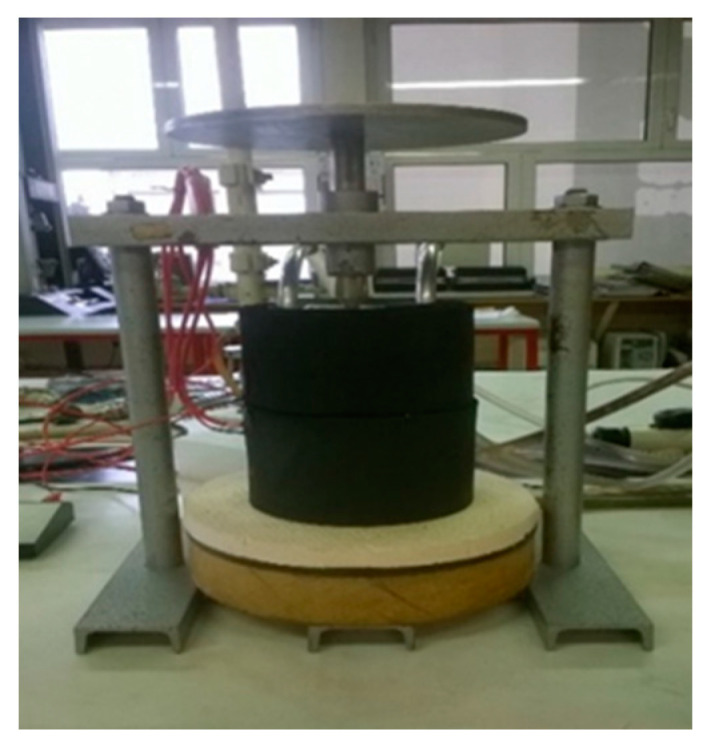
Apparatus for measuring the thermal conductivity.

The thermal conductivity was measured on an apparatus (Figure 2) which has an insulating plate at the bottom, on which we placed a warm block with a temperature of 60 °C. The temperature of the block is 60 °C. Then we placed a second copper measuring plate, a glass plate of known thermal conductivity (λ_x_ = 1.0319 Wm^−1^K^−1^) and a thinner copper measuring plate. This was followed by our sample, a thicker measuring plate and a block of 20 °C temperature. We connected everything together with thermocouples to a temperature meter. The temperatures were measured for three minutes until the individual values remained unchanged with repeated measurements [11,23,28,29,30,31].

Equation (2) was used to calculate the thermal conductivity:

(2) $$\lambda_{x}=\lambda_{n}\cdot \frac{dx}{dn}\cdot \frac{T_{3}-T_{2}}{T_{2}-T_{1}}$$

where *λ_x_* is the thermal conductivity of the test piece (Wm^−1^K^−1^), *λ_n_* is the thermal conductivity of the reference glass pane (*λ_n_* = 1.0319 Wm^−1^K^−1^), *dx* is the thickness of the test piece (mm), *T*_1_ is the temperature of the thin copper plate (K), *T*_2_ is the temperature of the middle copper plate (K) and *T*_3_ is the temperature of the thicker copper plate (K).

#### 3.2.5. Water Vapor Permeability

The water vapor permeability was measured according to the ASTM E96:E96M [32] standard. For the measurement of water vapor permeability, glass containers with a metal lid that had a 3 cm diameter opening in the center were used, and 7 mL of distilled water was pipetted into a beaker. The sample was placed face down on the jar, covered with a lid and fixed. The sample dishes were weighed after one hour and then placed in a chamber for 24 h. The conditions in the chamber were as follows: RH = 55%, T = 23 °C [11,15,18,28,29,30].

#### 3.2.6. Functionality of Nonwoven Insulating Layer

Nonwovens as insulating layers are ideal for providing barrier properties in protective gloves where such properties are desired. Structured nonwovens provide a large surface area for the entrapment of any number of substances that might be harmful to the wearer. For optimum protection, nonwoven fabrics are often used in combination with outer and inner layers with a microporous membrane as the second inner layer. The inner nonwoven layer(s) may provide insulation of the multilayered structure and also other important comfort properties such as air permeability and water vapor permeability. Depending on its thickness, the outer layer also contributes to the strength of the structure so that the fabric maintains its integrity through construction processes and wear.

Depending on the application, the inserted microporous membrane should be impermeable to chemicals, bodily fluids, infectious agents or other materials. On the other hand, the microporous membrane is permeable to air and water vapor.

To produce a surface structure with a velour or ribbed effect, structuring looms (also called fork needles) are used. Instead of carrying fibers into the hole in the base plate, fork needles carry tufts into lamellar bars that extend from the entrance to the exit of the needle loom. These fork needles carry large fiber tufts into parallel lamellar bars. These bars carry the fiber tuft from the input to the output side of the needling machine. The so-called structured surface of the nonwoven produces influences on the functional properties such as mechanical, insulation and permeability properties [7,8,9,10,11,12,21,22,23,24,28,29,30,31,33,34,35,36].

#### 3.2.7. Statistical Analysis

The impact of the technology of the needling (flat base plate and lamellar base plate) process on the functional properties of analyzed fabrics (breaking force and elongation, elastic recovery after compressive loading, air permeability, thermal conductivity, water vapor permeability) was tested using analysis of variance (ANOVA) to determine the significance of technology of needling process and incorporation of a microporous membrane on functional properties of three-layered samples which are used as an insulating layer for protective gloves.

The basis of a one-factor ANOVA is represented by the partitioning of the sums of squares into between-class (SSb) and within-class (SSw). This technique enables all of the classes to be compared with each other simultaneously, rather than individually. This method also assumes that the samples are normally distributed. The one-factor analysis is calculated in three steps. The sums of squares are determined first for all of the samples and then for the within-class and between-class cases. For each stage, the degrees of freedom (df) are determined as well, where df is the number of independent “pieces of information” involved in the estimate of a parameter. These calculations are used with the Fisher statistics to analyze the null hypothesis. The null hypothesis states that there are no differences between the means of different classes, suggesting that the variance of the within-class samples should be identical to that of the between-class samples. If the calculated F-ratio in a test is larger than the F-critical value, then the null hypothesis is rejected. These results are then tested for statistical significance or *p*-value, where the *p*-value is the probability that a variate assumes a value greater than or equal to the value observed strictly by chance. If the *p*-value is low (i.e., *p* ≤ 0.05 or *p* ≤ 5%), then the null hypothesis is rejected, indicating that differences exist between the classes and that these differences are statistically significant. If the *p*-value is greater than 0.05 (i.e., *p* > 0.05 or *p* > 5%), then the null hypothesis is accepted, indicating that the differences between classes are accidental. If the *p*-value is equal 0.05 or lower, the result is considered significant, and the null hypothesis is rejected, indicating that differences exist between the classes and that these differences are statistically significant. 

ANOVA was performed using the SPSS Statistics software (IBM, London, UK) [37].

## 4. Results and Discussion

### 4.1. Results of Breaking Stress and Elongation 

The results of breaking stress and elongation are presented in Table 4.

The highest breaking elongation in both directions is displayed by the sample ST. The longitudinal elongation at break is 98.13% and 42.57% in the transverse direction. The highest degree of breaking elongation of the sample ST is due to the bonding using a fork needle on the lamellar base plate, which affects the change in the structure of the base layer and thus the increase in breaking elongation (the samples ST and STL have higher values of breaking elongation than the samples T and TL) (Table 4). The breaking elongation of the samples ST and STL reinforced on the lamellar base plate is higher than that of the samples T and TL reinforced on the flat base plate. The TL sample has a maximum breaking stress in the longitudinal direction of 1.272 N/mm^2^ and in the transverse direction of 0.735 N/mm^2^, followed by the STL sample with a breaking stress of 1.127 N/mm^2^. It follows that the TL and STL specimens containing a microporous PES membrane have a higher breaking stress than the T and TL samples without microporous membranes (Table 4).

The statistical analysis using one-way ANOVA shows the statistically significant influence of the technology of the needling process on the breaking elongation (*p*_value_ < 0.05). On the other hand, the statistical analysis shows the statistically non-significant influence of the technology of the needling process and the incorporation of the microporous membrane on the breaking stress.

### 4.2. Results of the Yield Point and Elasticity Modulus 

The results of the yield point and the elasticity modulus are shown in Table 5.

The maximum elongation at the yield point in the longitudinal direction is displayed by the STL sample (39.05%). The stress at the yield point is also highest for the STL sample at 0.229 N/mm^2^. The elongation at the yield point is highest for the ST and STL samples, both in the longitudinal and transverse directions. The reason for the higher elongation is due to the structured web surface of samples ST and STL (Table 5) compared to samples T and TL (Table 5), where the web is bonded on the flat base plate. At the same time, the samples ST and STL have the lowest elasticity modulus, which means that they are less resistant to further loading, which affects the higher value of elongation at E_o_ in the range of elasticity (20%) and finally the breaking elongation in the longitudinal direction. STL is also the highest (Table 5). The higher breaking elongation and yield point elongation and lower elasticity modulus are influenced by the structured surface of the web which was needle-bonded using fork needles on the lamellar base plate, which affects the higher degree of web elongation.

The statistical analysis using one-way ANOVA shows the statistically non-significant influence of the needling technology process on the elasticity modulus (*p*_value_ > 0.05). The statistical analysis also shows the statistically non-significant influence of the microporous membrane on the elasticity modulus.

### 4.3. Results of Elastic Recovery after Compression Loading

**Figure 3:**
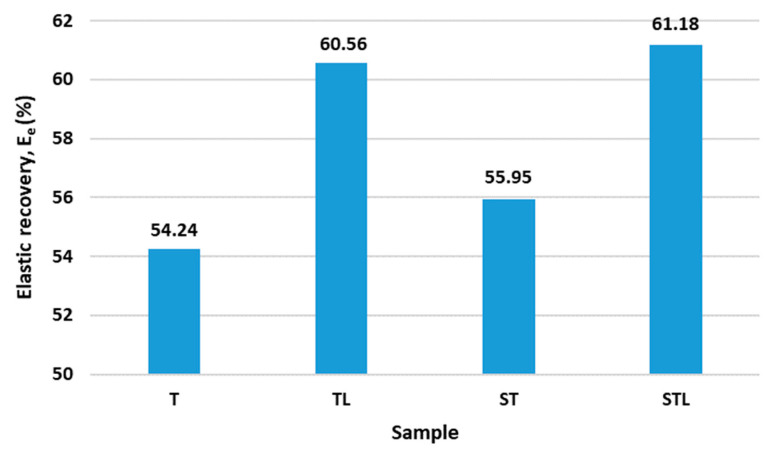
The results of elastic recovery after compression loading.

The results of the elastic recovery after compression loading are shown in Figure 3.

The highest elastic recovery after compressive loading, which is 61.18%, is observed for the STL sample. This is followed by the TL sample with an elastic recovery of 60.56 %. Both samples contain a microporous PES membrane which leads to an increase in elastic recovery. Consequently, samples T and ST, which do not contain a microporous membrane, have a 10% lower elastic recovery. The STL sample also has the highest elongation at the yield point (39.05%), which means that it also has a larger area where the deformations are fully reversible. The same is true for the TL sample, which has an elongation at the yield point value of 10.00%. Both samples (TL and STL) have a microporous membrane with a thickness of 20 µm (Figure 3).

Statistical analysis using one-way ANOVA shows the statistically non-significant influence of the needling technology process on elastic recovery after compression loading (*p*_value_ > 0.05). On the other hand, the statistical analysis shows a statistically significant influence of the microporous membrane on the elastic recovery after compression loading.

### 4.4. Results of the Permeability Properties 

#### 4.4.1. Water Vapor Permeability

**Figure 4:**
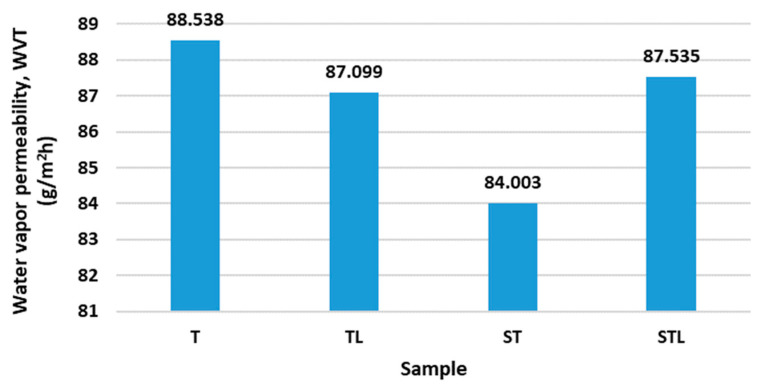
The results of water vapor permeability.

Figure 4 shows the results of water vapor permeability.

Sample T has the highest water vapor permeability. Samples T, TL and STL have similar vapor permeability values (from 87.009 to 88.538 g/m^2^h). A greater change in water vapor permeability is shown by sample ST (84.003 g/m^2^h), which does not have a microporous PES membrane inserted and has a structured nonwoven surface, which most likely decreases water vapor permeability because it is more difficult for water vapor to pass through the structured fiber surface into the environment. Higher water vapor permeability is achieved on average by samples T and TL, which are needle-bonded on the flat base plate, while water vapor permeability does not increase even with a microporous PES membrane (sample TL) inserted, which was expected. Thus, the microporous membrane has an effect on the higher water vapor permeability only for sample STL, which is bonded to the lamellar base plate and has a structured surface. There are larger differences in water vapor permeability between the ST and STL samples (84.003 g/m^2^h and 87.535 g/m^2^h) (Figure 4).

Statistical analysis using one-way ANOVA shows a statistically non-significant influence of the needling technology process and microporous membrane on the water vapor permeability (*p*_value_ > 0.05).

#### 4.4.2. Air Permeability

**Figure 5:**
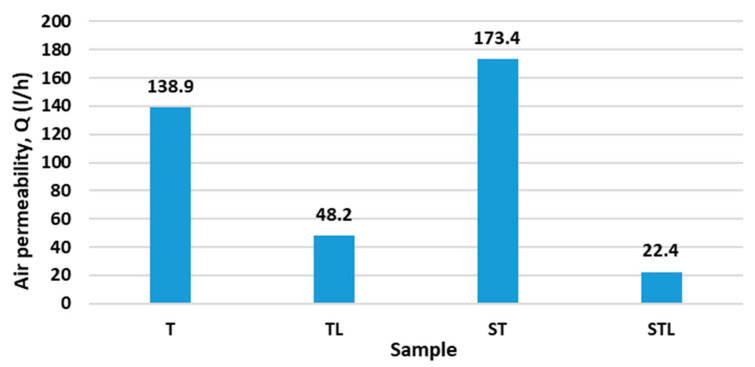
The results of air permeability.

Figure 5 shows the results of air permeability.

The sample ST has the highest air permeability, followed by sample T, which has no microporous membrane inserted. The air permeability value for samples T and ST are 138.9 L/h and 173.41 L/h, respectively. For the TL and STL samples, which contain a microporous PES membrane, the air permeability is reduced by more than 50%, ranging from 22.4 L/h for the STL sample to 48.2 L/h for the TL sample. The lowest air permeability was measured for the STL sample (22.4 L/h), which contains a microporous PES membrane and has a structured surface, affecting the lower air permeability. Thus, the air permeability of the samples is significantly reduced in the STL sample, which contains a microporous PES membrane and has a structured nonwoven surface, which significantly affects the air permeability of the sample. The samples T and TL, which have a flat surface (needle-bonded on the flat base plate), also have higher values of air permeability on average, in the cases with and without inserted microporous PES membrane (Figure 5).

The statistical analysis using one-way ANOVA shows the statistically non-significant influence of the technology of the needling process on the air permeability (*p*_value_ > 0.05). On the other hand, the statistical analysis shows a statistically significant influence of the incorporation of the microporous PES membrane on air permeability.

#### 4.4.3. Thermal Conductivity

**Figure 6:**
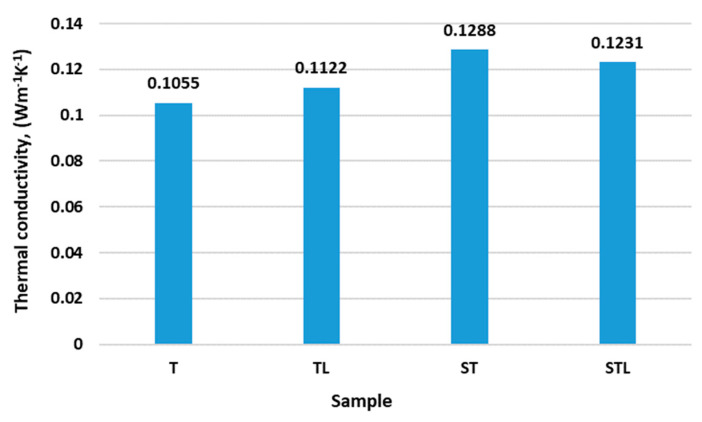
The results of thermal conductivity.

Figure 6 shows the results of thermal conductivity.

The results of the thermal conductivity analysis show that the sample ST (0.1288 Wm^−1^K^−1^) has the highest thermal conductivity, followed by the sample STL (0.1231 Wm^−1^K^−1^). The samples ST and STL consist of fibers of 100% PES, which have higher thermal conductivity than samples T and TL (0.1055 Wm^−1^K^−1^ and 0.1222 Wm^−1^K^−1^), which consist of a mixture of PES/CV/WO, which has lower thermal conductivity than 100% PES fibers. The samples T and TL have on average a lower thickness than the samples ST and STL, but the raw material composition has in this case a greater influence on the thermal conductivity of the fiber than the structure of the nonwoven web. The microporous PES membrane has an influence on the increase in thermal conductivity (0.1122 Wm^−1^K^−1^) only for the TL sample, while it slightly decreases for the STL sample. The ST and STL samples, which have a structured surface (needle-bonded on the lamellar base plate), have higher thermal conductivity, but the inserted microporous PES membrane has no significant effect on the change in thermal conductivity in the STL sample (relative to the ST sample).

For protective gloves, the T sample is the most suitable, having the lowest thermal conductivity and therefore the best insulating properties compared to the other samples analyzed (Figure 6).

The statistical analysis using one-way ANOVA shows the statistically non-significant influence of the technology of the needling process and incorporation of a microporous membrane on the thermal conductivity (*p*_value_ > 0.05).

#### 4.4.4. The Statistical Analysis

One-way ANOVA was used to determine the significance of the influence of the technology of the needling process and incorporation of a microporous membrane on the functional properties of three-layered samples for protective gloves (*p*_value_ < 0.05) (Table 6).

## 5. Conclusions

In the presented research, we investigated three-layer samples used for protective gloves, where the inner layer consists of a mixture of PES/CV/WO fibers (samples T and TL) and has a smooth surface and uniform structure, while the TL sample has a microporous PES membrane. The inner layer of samples ST and STL consists of a structured nonwoven of 100% PES fibers, and a microporous PES membrane is inserted in the STL sample.

Based on the studies of the functional properties of protective gloves, it can be argued that the structural needling of the lamellar base layer has an effect on greater breaking elongation. The samples ST and STL, which have a structured surface, have the highest elongation. The higher breaking elongation is also influenced by the inserted microporous PES membrane in the samples TL and STL. Samples TL and STL with the microporous PES membrane also have higher breaking stress than samples T and ST.

The stress and elongation at the yield point (elastic limit) are also higher in the ST and STL samples, which have a structured surface as the consequence of needle bonding on the lamellar base plate. The elasticity modulus of the ST and STL samples is the lowest, which means that they are poorly resistant to further loading, and the structured surface of the samples also affects the higher values of breaking elongation. The inserted microporous membrane influences the higher values of stress and elongation at the yield for the STL sample, which has a structured surface (needle-bonded on a lamellar base plate) with an inserted microporous PES membrane.

Higher values of elastic recovery under compressive loading are influenced by the structured surface of the nonwoven of the STL sample, which is needle-bonded on the lamellar base plate. The inserted microporous membrane of the TL and STL samples influences the better elastic properties of the nonwovens and on the other hand the lower air permeability (samples TL and STL).

For the TL and STL samples with the microporous PES membrane, the air permeability is reduced by more than 50% and ranges from 22.4 L/h for the STL sample to 48.2 L/h for the TL sample.

The ST and STL samples have the highest thermal conductivity, with the fiber consisting of 100% PES fibers. Samples T and TL, whose fibers consist of a mixture of PES/CV/WO fibers, have lower thermal conductivity than samples ST and STL. In this study, the raw material composition before sample construction had a greater influence on thermal conductivity. Samples T and TL are more suitable as an insulating layer for protective gloves in terms of thermal conductivity.

From the results of the presented research, it can be concluded that the content of the microporous membrane improves the mechanical properties and elastic properties of the samples. On the other hand, the incorporation of a microporous membrane causes lower air permeability and higher water vapor permeability, as in the case of the STL sample which contains a microporous membrane and has a nonwoven with a structured surface. The water vapor permeability is higher in the case of samples with a smooth, flat surface, but it is greatly increased in the case of the STL sample containing a microporous membrane and having a nonwoven with a structured surface.

The structured nonwoven in the ST and STL samples affects the higher thermal conductivity of the samples.

The results of the analysis showed that the thermal conductivity of the samples is significantly influenced by the raw material composition of the web rather than the structure of the web.

## Figures and Tables

**Table 1:** Raw material composition of analyzed samples.

Sample	Raw Material				Microscope View of Microporous Membrane 70× Magnitude
	1st Layer	2nd Layer	3rd Layer	Microporous Membrane	
T	CO	PES/CV/WO	CO/PET	/	
TL	CO	PES/CV/WO	CO/PET	PES	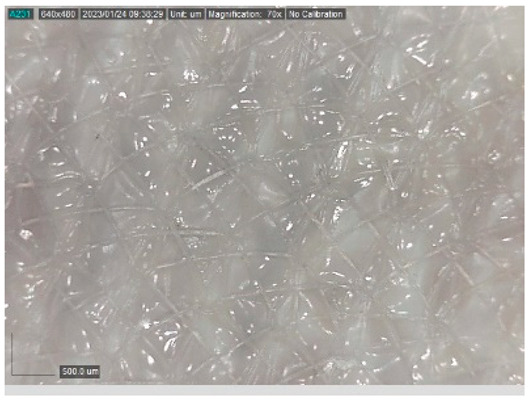
ST	CO	PES	CO/PET	/	
STL	CO	PES	CO/PET	PES	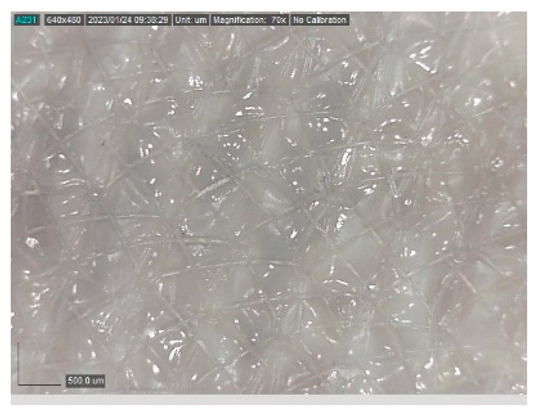

**Table 2:** Structural properties of the samples.

Sample	Mass, M (g/m^2^)	Thickness, h (mm)	Mass of Nonwoven, M_nonwoven_ (g/m^2^)	Thickness of Nonwoven, d_nonwoven_ (mm)	Diameter of Fibers in Nonwoven, d_fibers_ (µm)		
					PES	CV	Wo
**T**	1367.3	8.416	637.7	4.456	26.0	18.0	42.0
**TL**	1427.6	8.350	610.1	4.391	28.0	18.0	45.0
**ST**	1195.8	9.407	307.9	5.301	25.0	-	-
**STL**	1278.1	9.439	336.9	5.285	29.0	-	-

**Table 3:** Microscopic view of the samples.

Sample	1st Layer	2nd Layer	3rd Layer
**T**	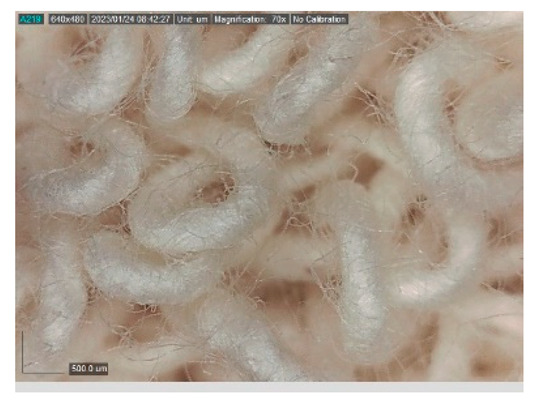	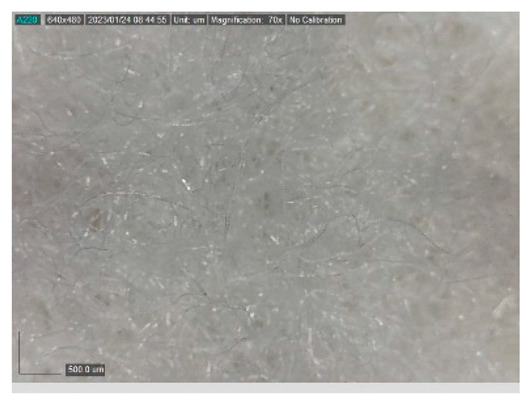	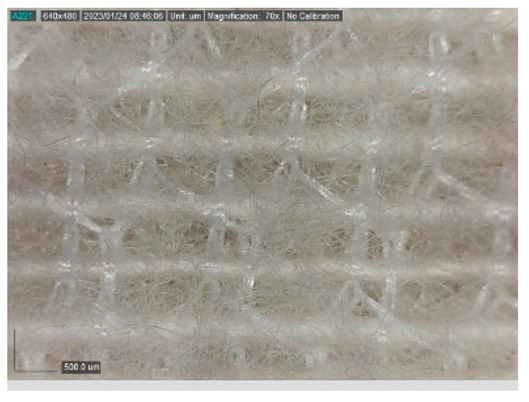
**TL**	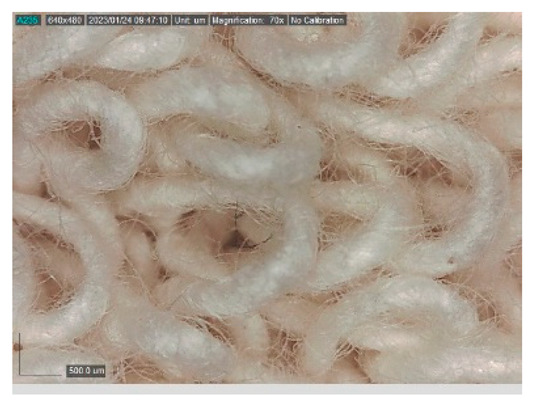	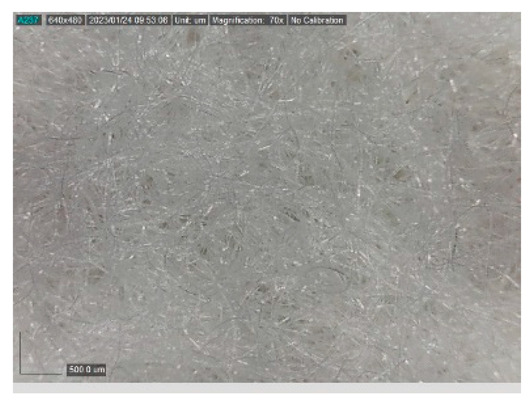	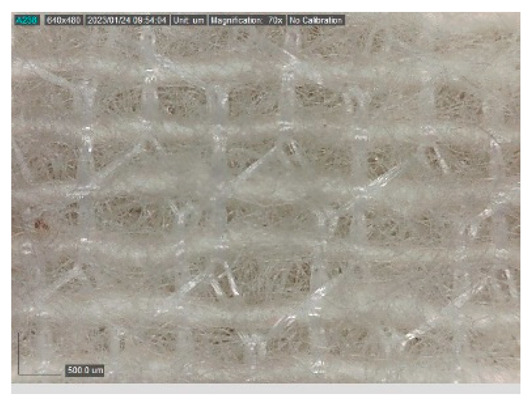
**ST**	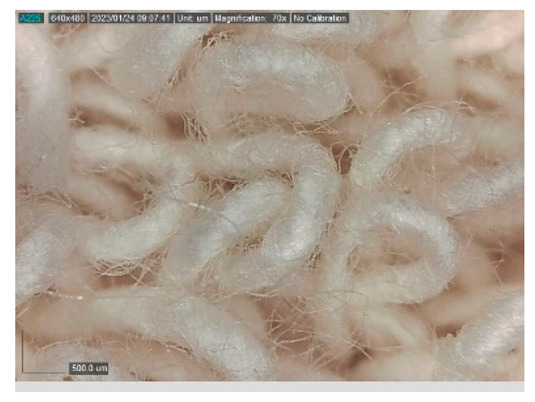	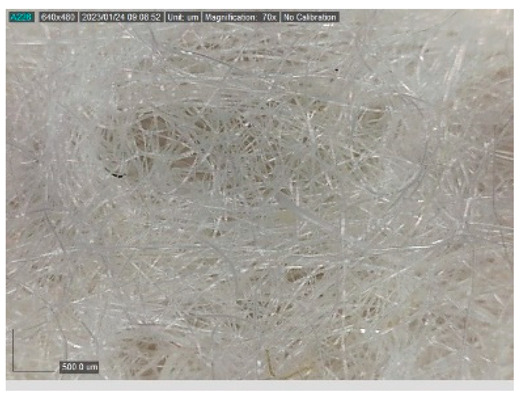	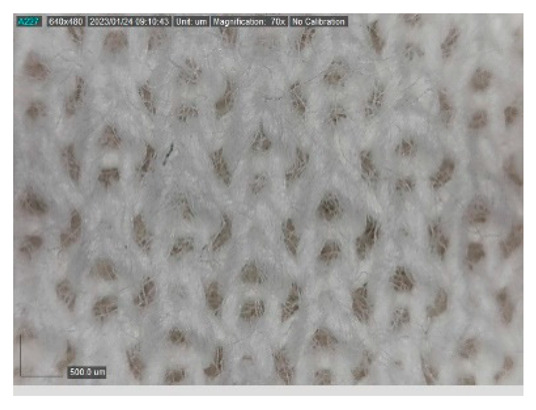
**STL**	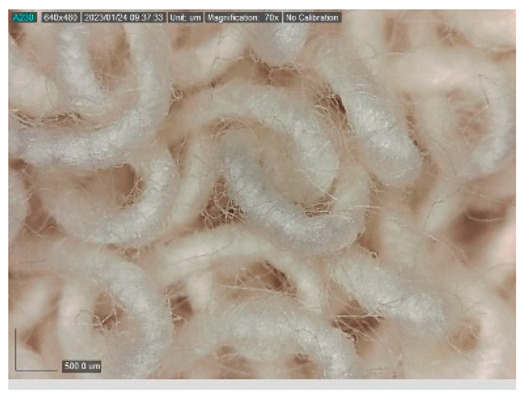	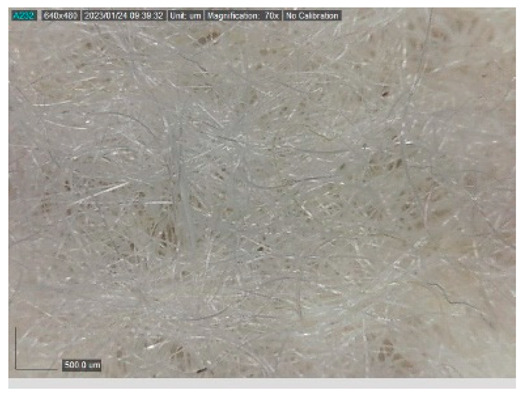	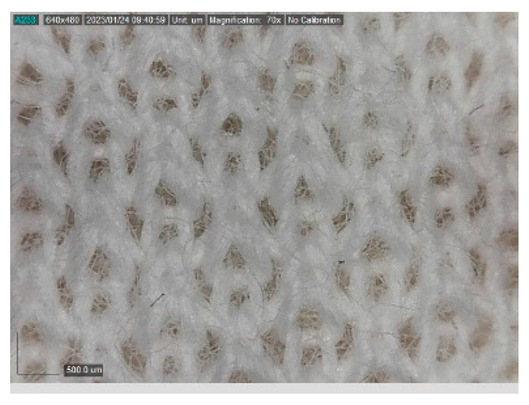

**Table 4:** Breaking stress and elongation of the analyzed samples.

Sample	Breaking Elongation, ε (%)		Breaking Stress, σ (N/mm^2^)	
	Longitudinal	Transverse	Longitudinal	Transverse
**T**	11.02	28.55	0.724	0.842
**TL**	37.05	24.04	1.272	0.735
**ST**	98.13	42.57	0.900	0.742
**STL**	94.12	39.05	1.127	0.824

**Table 5:** The results of yield point and elasticity modulus.

Sample	Elongation at the Yield Point, ε_y_ (%)		Stress at the Yield Point, σ_y_ (N/mm^2^)		Elasticity Modulus, E_o_ (N/mm^2^)		Elongation at E_o_ (%)	
	Longitudinal	Transverse	Longitudinal	Transverse	Longitudinal	Transverse	Longitudinal	Transverse
**T**	10.0	6.7	0.25	0.203	0.056	0.024	4.0	2.0
**TL**	10.0	7.0	0.15	0.232	0.012	0.027	4.0	3.0
**ST**	36.05	16.58	0.119	0.099	0.002	0.004	20.0	7.71
**STL**	39.05	14.0	0.229	0.198	0.005	0.014	21.0	6.0

**Table 6:** Results of one-way ANOVA.

**ANOVA for Breaking Stress Results**						
***Source of Variation***	***SS***	***df***	***MS***	***F***	***p-Value***	***F-Crit***
Between Groups (technology of needling process)	0.0005	1	0.0005	0.00106	0.975	5.987
Between Groups (without or with microporous membrane)	0.070	1	0.070	1.992	0.208	5.987
**ANOVA for Breaking Elongation Results**						
***Source of Variation***	***SS***	***df***	***MS***	***F***	***p-Value***	***F-Crit***
Between Groups (technology of needling process)	3750.2	1	3750.2	6.566	0.0427	5.987
Between Groups (without or with microporous membrane)	24.465	1	24.465	0.0205	0.891	5.987
**ANOVA for Elasticty Modulus Results**						
***Source of Variation***	***SS***	***df***	***MS***	***F***	***p-Value***	***F-Crit***
Between Groups (technology of needling process)	0.0011	1	0.0011	5.867	0.051	5.987
Between Groups (without or with microporous membrane)	9.8·10^−5^	1	9.8·10^−5^	0.275	0.619	5.987
**ANOVA for Elastic Recovery after Compression Loading Results**						
***Source of Variation***	***SS***	***df***	***MS***	***F***	***p-Value***	***F-Crit***
Between Groups (technology of needling process)	1.357	1	1.357	0.0806	0.803	18.512
Between Groups (without or with microporous membrane)	33.350	1	33.350	40.321	0.024	18.512
**ANOVA for Water Vapor Permeability Results**						
***Source of Variation***	***SS***	***df***	***MS***	***F***	***p-Value***	***F-Crit***
Between Groups (technology of needling process)	4.200	1	4.200	1.155	0.395	18.513
Between Groups (without or with microporous membrane)	1.095	1	1.095	0.211	0.691	18.513
**ANOVA for Air Permeability Results**						
***Source of Variation***	***SS***	***df***	***MS***	***F***	***p-Value***	***F-Crit***
Between Groups (technology of needling process)	18.922	1	18.922	0.00243	0.965	18.513
Between Groups (without or with microporous membrane)	14,604.72	1	14,604.72	31.478	0.030	18.513
**ANOVA for Thermal Conductivity Results**						
***Source of Variation***	***SS***	***df***	***MS***	***F***	***p-Value***	***F-Crit***
Between Groups (technology of needling process)	0.000292	1	0.000292	15.115	0.060	18.513
Between Groups (without or with microporous membrane)	2.5·10^−7^	1	2.5·10^−7^	0.00151	0.972	18.513

Sum-of-squares (SS) column with no repeated measures, degrees of freedom (df), mean squares (MS), F-ratio (F), *p*-value, F-critical (F-crit).

## Data Availability

The data presented in this study are available on request from the corresponding author.
